# Frailty and associated risk factors in patients with Sjögren’s disease: a cross-sectional study

**DOI:** 10.3389/fimmu.2025.1689685

**Published:** 2025-12-19

**Authors:** Zhen Tian, Qiuju Liao, Chunxiu Wang, Leiming Wang, Jagadish K. Chhetri, Yi Zhao, Piu Chan

**Affiliations:** 1 Department of Rheumatology and Allergy, Xuanwu Hospital of Capital Medical University, Beijing, China; 2 National Clinical Research Center for Geriatric Diseases, Advanced Innovation Center for Human Brain Protection, Capital Medical University, Beijing, China; 3 Evidence-based Medicine Centre, Xuanwu Hospital of Capital Medical University, Beijing, China; 4 Department of Neurobiology, Neurology and Geriatrics, Xuanwu Hospital of Capital Medical University, Beijing Institute of Geriatrics, Beijing, China

**Keywords:** cross-sectional study, frailty, inflammation, risk factors, Sjögren’s disease

## Abstract

**Introduction:**

This study aimed to evaluate the prevalence of frailty and to identify its associated risk factors in hospitalized patients with Sjögren’s disease (SjD).

**Methods:**

A cross-sectional study was conducted among hospitalized SjD patients at Xuanwu Hospital between August 2022 and October 2024. Frailty was evaluated using the Fried Frailty Phenotype, which comprises five components: unintentional weight loss, self-reported exhaustion, low physical activity, slowness, and weakness. Based on established criteria, patients were categorized as frail (≥3 criteria), pre-frail (1–2 criteria), or robust (0 criteria).

**Results:**

A total of 180 patients were included in the final analysis. The prevalence of frailty and pre-frailty was 27% and 49%, respectively. Multivariate logistic regression analyses identified higher c-reactive protein (OR = 1.080, 95% CI: 1.020-1.144, P = 0.008), the EULAR Sjögren’s Syndrome Disease Activity Index (OR = 1.082, 95% CI: 1.027-1.140, P = 0.003), and EULAR Sjögren’s Syndrome Patient Reported Index (OR = 1.271, 95% CI: 1.064-1.518, P = 0.008) as independent risk factors for frailty.

**Conclusions:**

Frailty is commonly observed among hospitalized patients with SjD and is independently associated with systemic inflammation and disease activity. These findings underscore the need for routine frailty assessment in clinical practice, particularly among patients with elevated inflammatory markers and more severe disease manifestations.

## Introduction

Sjögren’s disease (SjD) is a chronic inflammatory autoimmune disorder characterized by lymphocytic infiltration and progressive dysfunction of exocrine glands. While sicca symptoms remain the hallmark of the disease, extraglandular organ involvement is observed in over 40% of patients, contributing to its heterogeneous clinical presentation (1). SjD predominantly affects women over the age of 50, with a reported female-to-male ratio of approximately 14:1 (2, 3). Beyond the physical symptoms, SjD has been shown to significantly impair health-related quality of life (HR-QOL), reduce work productivity, and limit functional independence. Compared with the general population, individuals with SjD report lower SF-36 scores, decreased employment rates, and increased disability levels (4).

The long-term burden of systemic inflammation, compounded by chronic glucocorticoid use, may predispose patients to muscle atrophy, metabolic dysregulation, and declining physical function (5). These factors collectively increase susceptibility to frailty, a multidimensional syndrome defined by diminished strength, endurance, and physiological reserve, resulting in impaired resilience to external stressors (6). In the context of SjD, frailty is an emerging clinical concern due to its association with adverse outcomes such as increased hospitalization rates, greater healthcare utilization, and elevated economic burden.

Mounting evidence suggests that chronic low-grade inflammation—a hallmark of autoimmune diseases—plays a central role in the pathogenesis of frailty by promoting muscle catabolism, impairing immune and metabolic homeostasis, and accelerating biological aging (7–9). Clinical studies have demonstrated that frailty is strongly associated with increased risk of dependency, falls, mortality, impaired mobility, and disability progression (6, 10). Frailty has been extensively studied in other rheumatologic conditions, with reported prevalence rates ranging from 17–35% in systemic lupus erythematosus, 16.6% in rheumatoid arthritis, and 35.1% in systemic sclerosis (11–13). However, data on frailty in patients with SjD remain scarce. Given the characteristic fatigue, systemic manifestations, and functional impairment associated with SjD, this population may be particularly vulnerable to frailty. The lack of robust epidemiological data on frailty in SjD represents a critical gap in the current literature.

Therefore, the present study aimed to investigate the prevalence of frailty in hospitalized patients with SjD and to identify its associated risk factors. Understanding the determinants of frailty in this population may facilitate early identification, inform personalized management strategies, and ultimately improve long-term outcomes for individuals living with SjD.

## Materials and methods

### Study design and population

This cross-sectional study was conducted at Xuanwu Hospital, Capital Medical University (Beijing, China), in the Department of Rheumatology and Immunology from August 2022 to October 2024. The study adhered to the Strengthening the Reporting of Observational Studies in Epidemiology (STROBE) guidelines for cross-sectional research.

Patients diagnosed with SjD were consecutively recruited using a convenience sampling method to minimize selection bias. All eligible participants were hospitalized during the study period and provided written informed consent prior to enrollment. The study protocol was approved by the Ethics Committee of Xuanwu Hospital (Approval No. Clinical Research Review [055-001]) and was conducted in accordance with the Declaration of Helsinki and local institutional regulations.

### Inclusion and exclusion criteria

Inclusion criteria were as follows: (1) Age ≥18 years, regardless of gender (2) Fulfillment of the 2016 American College of Rheumatology/European League Against Rheumatism (ACR/EULAR) classification criteria for SjD (14) (3) Ability to provide written informed consent.

Exclusion criteria included: (1) Presence of acute infection (2) Diagnosis of malignancy within the past 5 years (3) Presence of other chronic inflammatory diseases (such as rheumatoid arthritis with concomitant SjD) (4) Inability to understand the informed consent due to language barriers or severe cognitive impairment (5) Incomplete clinical or assessment data.

All participants were hospitalized patients with SjD, representing a population with more severe disease and higher systemic inflammation compared to community-based cohorts.

### Frailty assessment

Frailty status were evaluated using the well-validated Fried Frailty Phenotype scale (6), which encompasses five key domains potentially influenced by chronic inflammation. Frailty assessment was performed during hospitalization following the standardized Fried criteria, classifying individuals as frail, pre-frail, or robust based on the presence of specific indicators.

The five frailty components were assessed as follows. First, unintentional weight loss was defined as self-reported weight loss of ≥4.5 kg or ≥5% of body weight within the past year. Second, exhaustion was assessed using two items from the Center for Epidemiologic Studies Depression Scale (CES-D) (15): “I felt that everything I did was an effort”, and “I could not get going.” Patients were asked to recall the frequency of these feelings during the past week. Response options were scored as follows: 0 = rarely or none of the time, 1 = some of the time (1–2 days), 2 = a moderate amount of time (3–4 days), and 3 = most of the time. A score of 2 or 3 on either item met the criterion for exhaustion.

Third, slowness was assessed based on height-stratified gait speed. A time of ≥6 seconds to walk 15 feet (approximately 4.5 meters) was considered slow for men with a height >173 cm or women >159 cm, and ≥7 seconds for men ≤173 cm or women ≤159 cm. Fourth, inactivity was evaluated using a simplified version of the Minnesota Leisure Time Activity Questionnaire, covering the frequency of participation in activities such as walking, housework, gardening, hiking, jogging, swimming, cycling, dancing, aerobics, and tennis. Fifth, weakness was measured using a digital handgrip dynamometer. Grip strength was tested three times in each hand, and the average value was used. Results were evaluated according to sex- and BMI-adjusted standards.

Based on these assessments, participants were classified as frail (meeting ≥3 criteria), pre-frail (1–2 criteria), or robust (0 criteria), in accordance with established guidelines.

### Data collection

Comprehensive demographic and clinical data were systematically collected from all enrolled patients through standardized case report forms. Demographic characteristics included age, sex, ethnicity, body mass index, smoking and alcohol history, living status, and education level. Low education level was operationally defined as high school or equivalent education and below. Clinical data collection encompassed disease-related information including disease duration and laboratory indicators, such as erythrocyte sedimentation rate (ESR), C-reactive protein (CRP), and rheumatoid factor (RF). All laboratory tests were performed using standardized protocols within the hospital’s central laboratory.

Disease activity evaluation employed two validated, complementary instruments. Disease activity was evaluated using the European League Against Rheumatism Sjögren’s syndrome disease activity index (ESSDAI) and the EULAR Sjögren’s Syndrome Patient Reported Index (ESSPRI) (16, 17). The ESSDAI instrument scores disease activity comprehensively across 12 systemic domains, with a total score range of 0–123 points; higher scores indicate greater disease activity. Based on ESSDAI scores, patients were categorized into three pre-defined groups: low disease activity (≤4 points), moderate activity (5–13 points), and high activity (≥14 points). The ESSPRI instrument assesses three subjective symptoms using visual analog scales (VAS): dryness, fatigue, and pain. Each symptom is scored from 0 to 10 points, with the final score calculated as the average of these three scores, reflecting the patient’s subjective symptom burden (18). Both assessments were performed by trained clinical staff to ensure consistency and accuracy.

Treatment history and comorbidity data were systematically documented. Current and previous treatment regimens were recorded, including glucocorticoid use (converted to prednisone equivalent dosage) and exposure duration, as well as the application of conventional disease-modifying antirheumatic drugs (cDMARDs). Comorbidity burden was assessed using the Charlson Comorbidity Index (CCI), which quantifies overall health status through weighted scoring based on the impact of 19 common comorbidities on mortality risk (19). All medication dosages were standardized and verified through medical records review.

Age categories were determined based on standard classifications commonly used in rheumatic disease research and clinical practice, in which adults aged 18–44 years are considered young, 45–59 years middle-aged, and ≥60 years older adults. Given that the age range of our study participants was 24–86 years, the final age groups were defined as 24–44 years (young), 45–59 years (middle-aged), and 60–86 years (older adults) for analysis.

### Sample size calculation

The sample size for this cross-sectional study was estimated based on the commonly accepted methodological standards for logistic regression analysis, which recommend 10–20 participants per predictor variable to ensure stable estimates. Given that 14 predictor variables were planned for inclusion in the multivariable logistic regression model, a minimum sample size of 140 to 280 participants was required. In the present study, a total of 180 participants were included, which exceeds the minimum recommended sample size and is sufficient for reliable multivariable analysis.

### Statistical analysis

Statistical analyses were conducted using SPSS version 26.0 (IBM Corp., Armonk, NY, USA). Categorical variables were summarized as frequencies and percentages (%), while continuous variables were presented as mean ± standard deviation (SD) or median with interquartile range (IQR), depending on the normality of their distribution. Normality was assessed using the Kolmogorov-Smirnov test for samples with n ≥ 50 and the Shapiro-Wilk test for smaller samples (n < 50). Group comparisons of categorical variables were conducted using the Chi-square test or Fisher’s exact test, as appropriate. Continuous variables were compared using the Mann-Whitney U test. To examine distributional trends of frailty phenotype across age groups (24-44, 45–59, 60–86 years) and disease activity levels (low, moderate, high), the Jonckheere-Terpstra test was applied.

Potential frailty risk factors were identified through binomial logistic regression: variables with P ≤ 0.1 in univariate analysis were screened; backward multivariate LR retained P < 0.05 variables; the final model included these plus age, a clinically relevant factor. Results (ORs, 95% CIs, P values) were shown in forest plots; collinearity diagnostics (VIFs) were performed, and sensitivity analyses were conducted using two added sets that incorporated different combinations of clinically relevant predictors; Two-sided p-values < 0.05 were considered statistically significant.

## Results

### Participant characteristics

**Figure 1:**
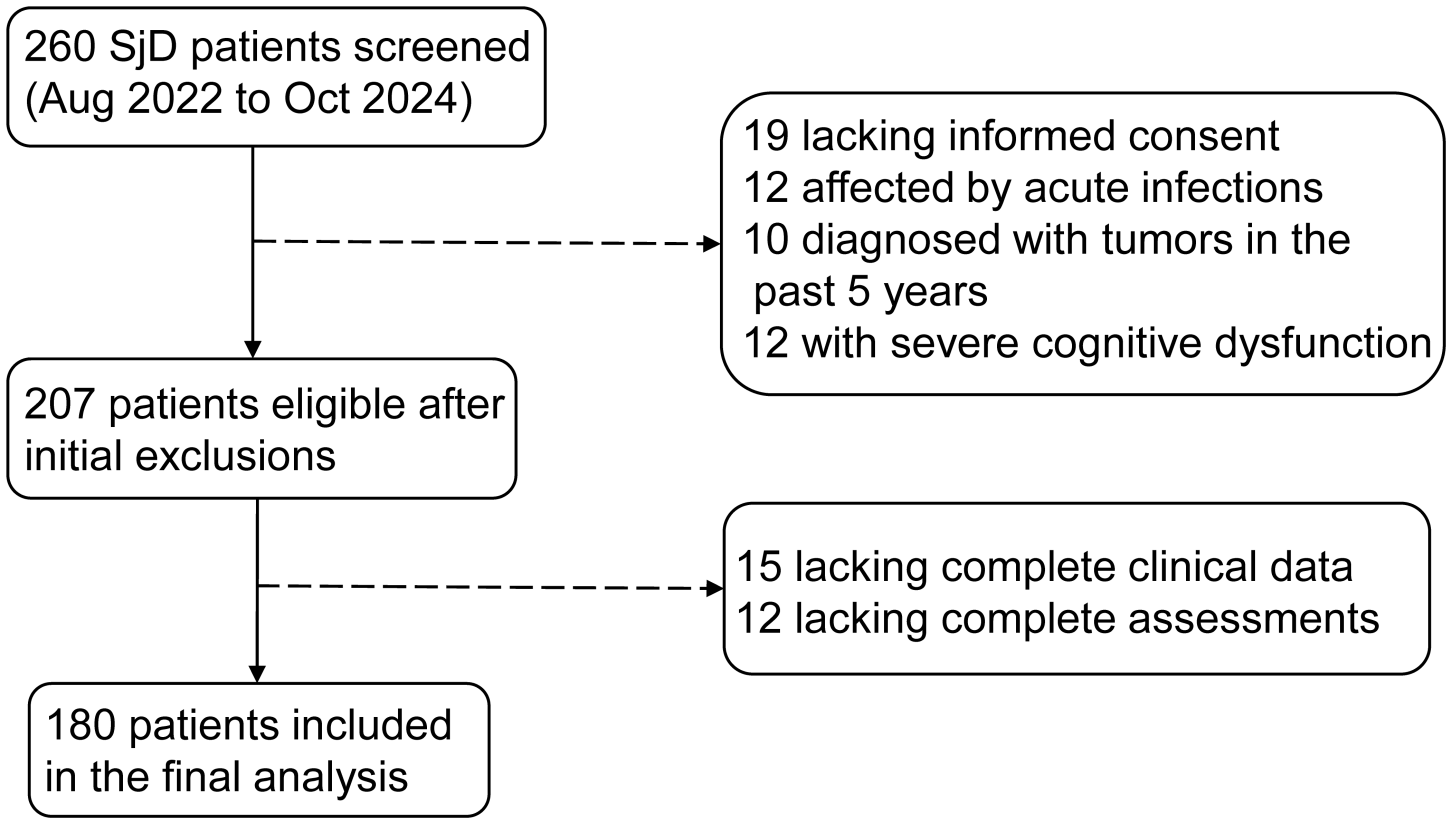
Patient enrollment flowchart. Flowchart showing the selection process of SjD patients. A total of 260 patients were initially screened, 53 were excluded due to various reasons, and 27 were excluded for incomplete data, resulting in 180 patients for final analysis.

A total of 260 SjD patients were initially screened for eligibility. After excluding 53 patients due to lack of informed consent, acute infection, recent malignancy, or severe cognitive impairment, 207 patients met inclusion criteria. Subsequently, 27 patients with incomplete data were excluded, resulting in a final sample of 180 patients for analysis (**Figure 1**). Baseline demographic and clinical characteristics are summarized in **Table 1**.

**Table 1:** Demographic and clinical characteristics of SjD Patients.

Characteristics	SjD patients (N = 180)
Age, years, median (IQR)	59.5 (51.0, 67.0)
Sex, female, n (%)	167 (92.8)
Han ethnicity, n (%)	167 (92.8)
BMI (kg/m2), mean (SD)	22.7 (3.5)
Ever smoked, n (%)	13 (7.2)
Ever used alcohol, n (%)	14 (7.8)
Living alone, n (%)	16 (8.9)
Low Education, n (%)	121 (67.2)
Disease duration, months, median (IQR)	60.0 (21.8, 120.0)
ESR (mm/h), median (IQR)	19.0 (10.0, 33.8)
CRP (mg/L), median (IQR)	2.0 (2.0, 4.0)
RF+, n (%)	84 (46.7)
ESSDAI, median (IQR)	5.0 (1.3, 12.0)
ESSPRI, median (IQR)	4.0 (3.0, 6.0)
Prednisolone-equivalent dose, mg/day, median (IQR)	0 (0, 15.0)
Glucocorticoid exposure duration, days, median (IQR)	1.0 (0, 6.8)
cDMARDs, n (%)	164 (91.1)
CCI, median (IQR)	1 (0,1)

BMI, body mass index; IQR, interquartile range; SD, standard deviation; ESR, erythrocyte sedimentation rate; CRP, C-reactive protein; RF, Rheumatoid Factor; ESSDAI, European League Against Rheumatism Sjögren’s Syndrome Disease Activity Index; ESSPRI, European Sjögren’s Syndrome Patient Reported Index; cDMARDs, conventional disease-modifying antirheumatic drugs; CCI, Charlson Comorbidity Index.

### Prevalence and distribution of frailty phenotype

**Figure 2:**
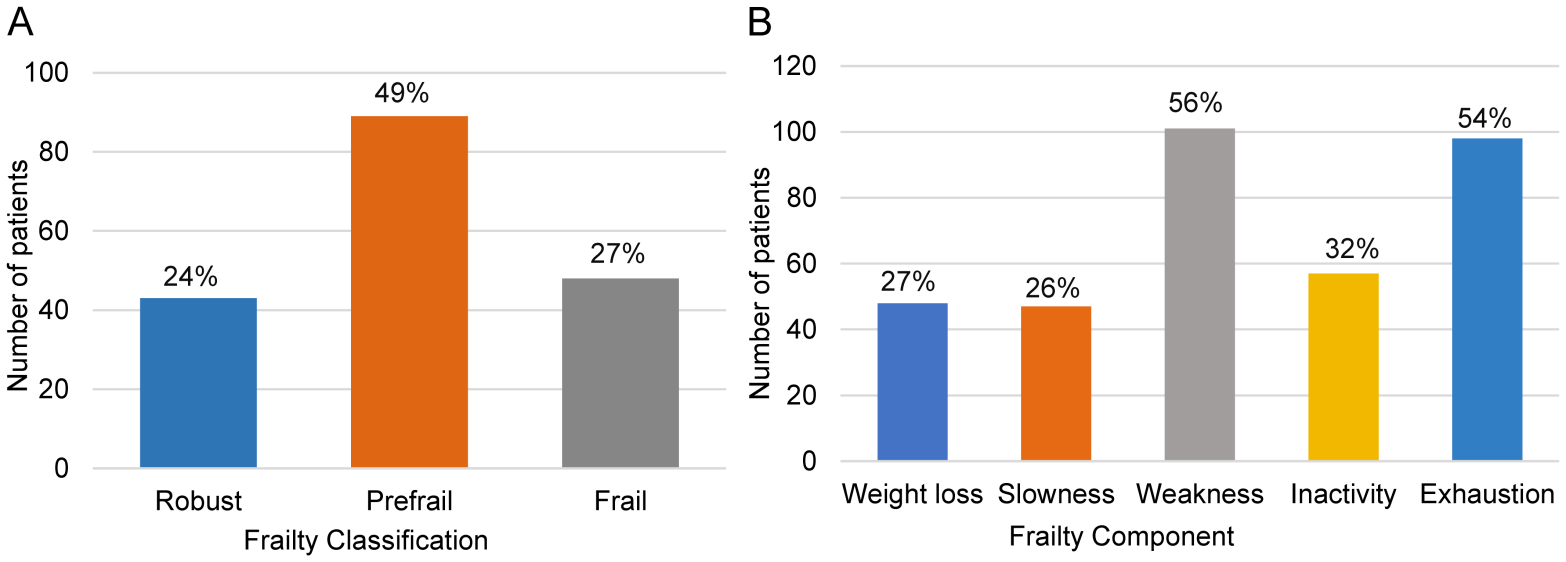
Frailty phenotype and components in patients with SjD. **(A)** Distribution of frailty classification based on Fried frailty phenotype showing 27% frail, 49% pre-frail, and 24% robust patients. **(B)** Frequency of individual frailty components, with weakness (56%) and exhaustion (54%) being the most common.

According to Fried’s frailty phenotype criteria, 26.7% (n=48; 95% CI: 20.2–33.1%) of patients were classified as frail, 49.4% (n=89; 95% CI: 42.1–56.8%) as pre-frail, and 23.9% (n=43; 95% CI: 17.3–30.6%) as robust, indicating that 76.1% of the cohort exhibited some degree of frailty (**Figure 2A**, **Supplementary Table S1**). Analysis of frailty components showed that weakness (56.1%) and exhaustion (54.4%) were the most prevalent features, whereas slowness was least common (26.1%) (**Figure 2B**, **Supplementary Table S1**).

### Association of frailty with demographic factors and disease activity

**Figure 3:**
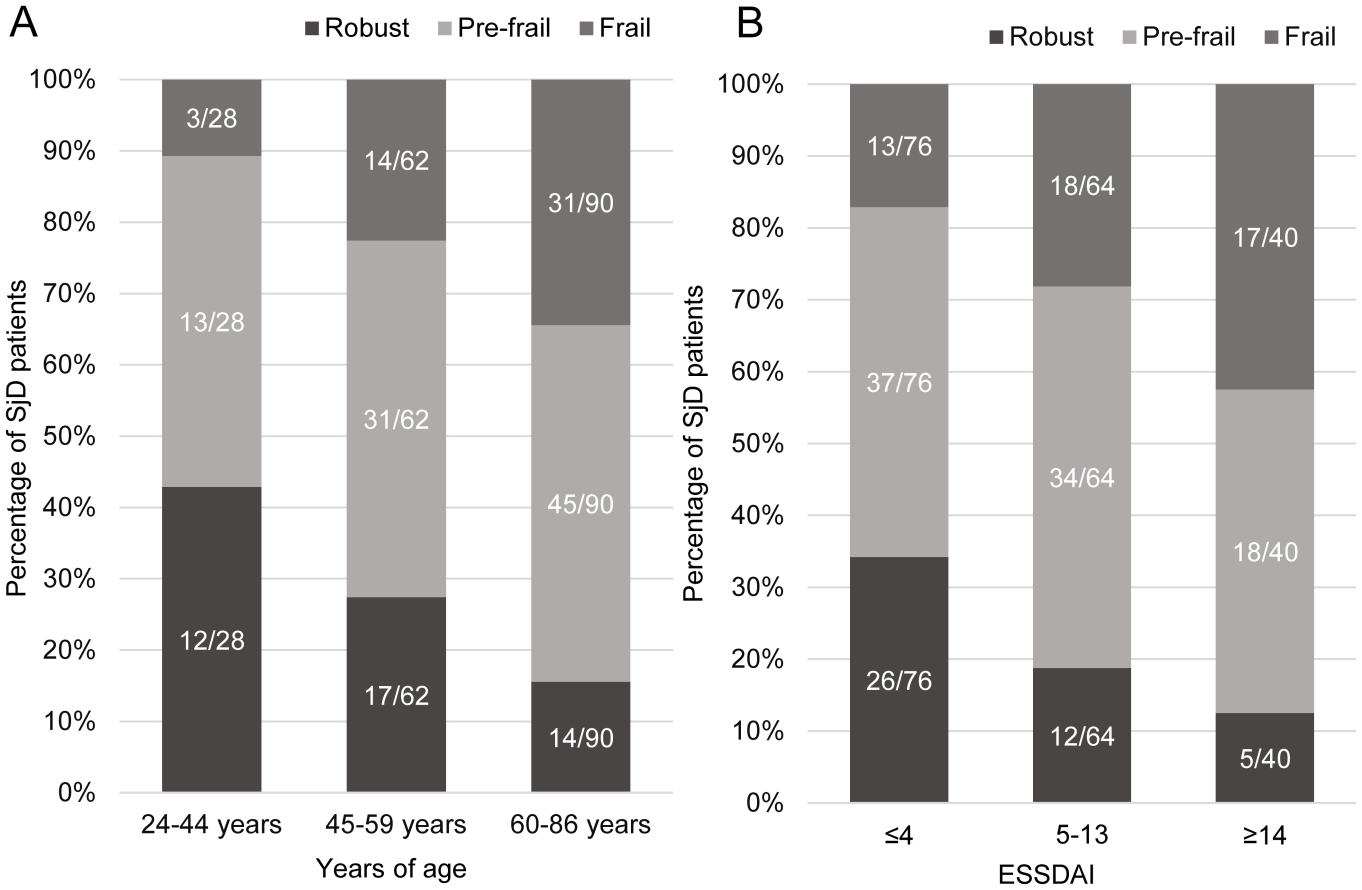
Distribution of Fried frailty phenotype by age group and disease activity. **(A)** Frailty prevalence across age groups (24-44, 45-59, 60–86 years), showing significant increase with age (p=0.001). **(B)** Frailty distribution across ESSDAI activity levels (low ≤4, moderate 5-13, high ≥14), demonstrating higher frailty prevalence with increased disease activity (p<0.001).

Frailty prevalence demonstrated a significant positive correlation with increasing age (p = 0.001), with rates of 10.7% in the youngest group (24–44 years), 22.6% in the middle-aged group (45–59 years), and 34.4% in the oldest group (60–86 years). Statistically significant differences were observed between the youngest and oldest groups (P = 0.002), while differences between the middle-aged and oldest groups approached significance (P = 0.063). No significant difference was found between the youngest and middle-aged groups (P = 0.135) (**Figure 3A**, **Supplementary Table S2**). Similarly, frailty prevalence increased significantly with higher disease activity as measured by ESSDAI scores (P < 0.001), with rates of 17.1%, 28.1%, and 42.5% in the low (ESSDAI ≤4), moderate (ESSDAI 5–13), and high activity (ESSDAI ≥14) groups, respectively. Significant differences were detected between the low and moderate (P = 0.039) and low and high activity groups (P = 0.001), while the difference between moderate and high activity groups was not statistically significant (P = 0.194) (**Figure 3B**, **Supplementary Table S3**).

### Comparison of characteristics between frail and non-frail patients

Participants were categorized into frail (n=48) and non-frail groups (n=132, including pre-frail and robust) for comparison (**Table 2**). Frail patients were older and more frequently had lower educational levels. They also demonstrated longer disease duration and higher levels of systemic inflammation, indicated by elevated CRP. Measures of disease activity (ESSDAI) and symptom burden (ESSPRI) were significantly greater in the frail group. Additionally, frail patients had higher cumulative prednisone doses and longer exposure times. Analyses using the three-tier frailty classification (frail, pre-frail, robust) yielded consistent patterns, with significant differences observed in CRP levels among groups (P = 0.009) (**Supplementary Tables S4, S5**).

**Table 2:** Demographic and Clinical Characteristics of Patients with Frailty and Non-frailty Phenotype.

Characteristics	Frail (n=48)	Non-frail (n=132)	P value
Age, years, median (IQR)	64.0 (54.0 70.0)	58.5 (49.3 66.0)	0.014
Sex female, n (%)	42 (87.5)	125 (94.7)	0.186
BMI, (kg/m²) mean (SD)	22.6 (4.1)	22.7 (3.3)	0.907
Ever smoked, n (%)	4 (8.3)	9 (6.8)	0.983
Ever used alcohol, n (%)	5 (10.4)	9 (6.8)	0.629
Living alone, n (%)	3 (6.3)	13 (9.8)	0.650
Low education, n (%)	38 (79.2)	83 (62.9)	0.040
Disease duration months, median (IQR)	84.0 (39.0 171.0)	45.0 (12.0 120.0)	0.003
ESR (mm/h), median (IQR)	21.5 (10.3 34.8)	18.0 (9.3 31.8)	0.164
CRP (mg/L), median (IQR)	3.0 (2.0 7.0)	2.0 (1.0 3.8)	0.003
RF+, n (%)	20 (41.7)	64 (48.5)	0.417
ESSDAI, median (IQR)	9.5 (4.0 16.5)	5.0 (1.0 11.0)	0.002
ESSPRI, median (IQR)	5.0 (4.0 7.0)	4.0 (2.3 5.0)	<0.001
Prednisolone-equivalent dose, mg/day median (IQR)	8.8 (0 30.0)	0 (0 15.0)	0.012
Glucocorticoid exposure duration, days median (IQR)	1.0 (0 204.3)	0 (0 1.0)	0.011
cDMARDs use, n (%)	44 (91.7)	120 (90.9)	1.000
CCI, median (IQR)	1 (0 2)	1 (0 1)	0.067

### Logistic regression analyses of frailty

**Figure 4:**
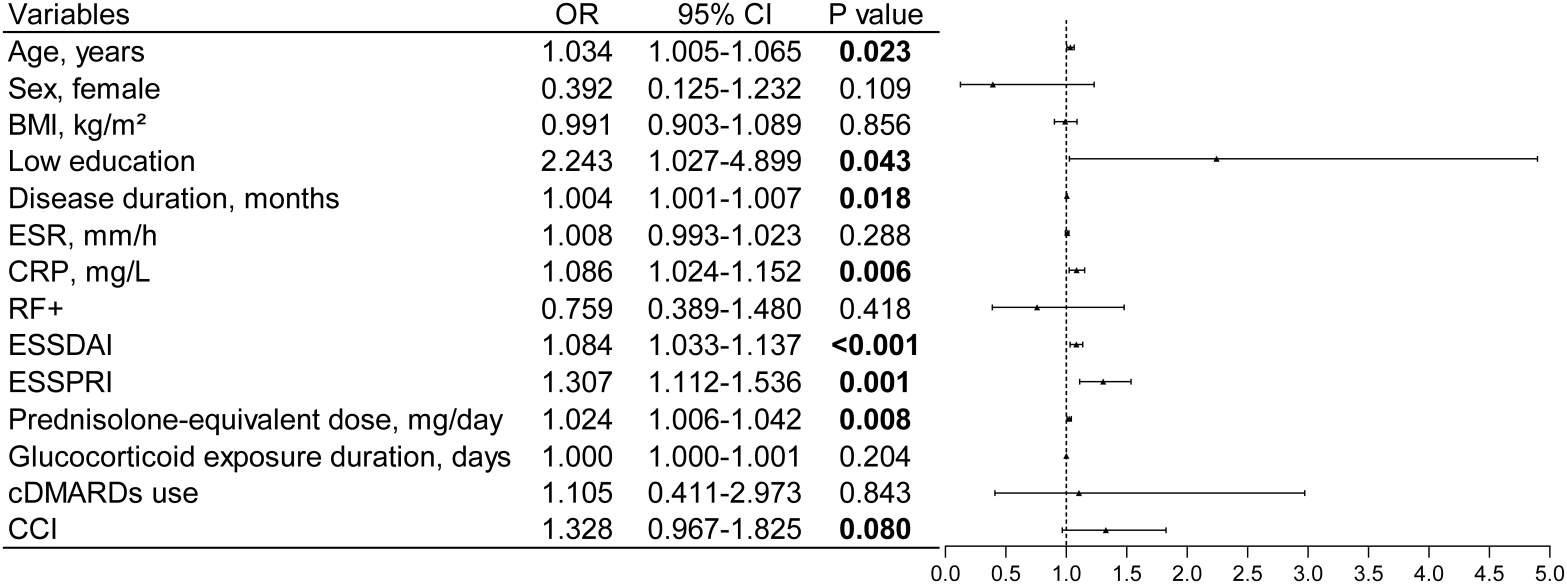
Univariate binary logistic regression for identifying factors associated with frailty. Variables with a P value ≤ 0.1 in the univariate analysis were included for further consideration. OR odds ratio; CI confidence interval.

**Figure 5:**
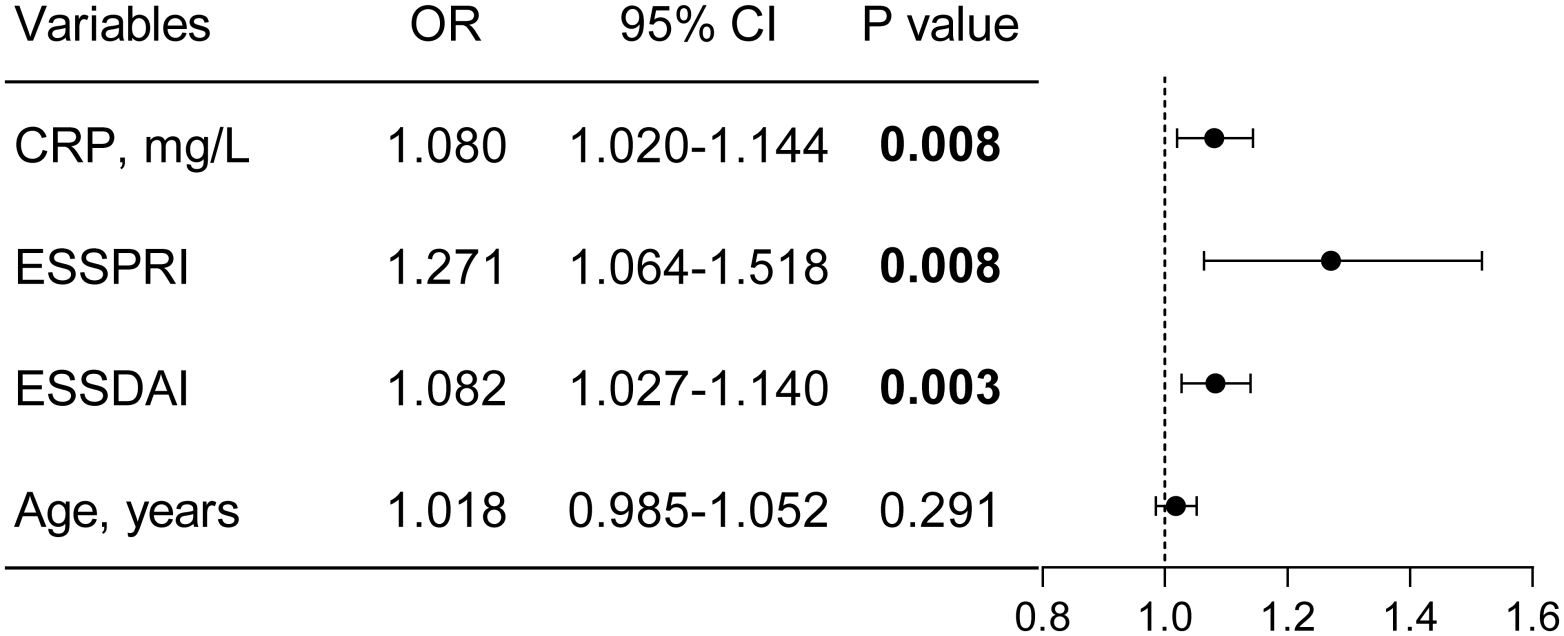
Independent risk factors for frailty identified by multivariable analysis.

After adjustment for confounders in multivariate analysis, only elevated CRP levels (OR 1.080; 95% CI, 1.020–1.144; P = 0.008), increased ESSDAI scores (OR 1.082; 95% CI, 1.027–1.140; P = 0.003), and higher ESSPRI scores (OR 1.271; 95% CI, 1.064–1.518; P = 0.008) remained independently associated with frailty (**Figure 4** and **Figure 5**). Collinearity diagnostics confirmed the absence of significant multicollinearity among variables included in the final model (**Supplementary Table S6**).

### Sensitivity analyses

Sensitivity analyses using alternative multivariable models yielded consistent results (**Supplementary Table S7**). In both Model 1 (adjusted for age, disease duration, CRP, ESSDAI, and ESSPRI) and Model 2 (further adjusted for low education), CRP, ESSDAI, and ESSPRI remained significantly associated with frailty. Age, disease duration, and low education were not significant predictors in either model. These findings confirm the robustness of the identified risk factors.

## Discussion

In this cross-sectional study, we comprehensively assessed the prevalence of frailty and its risk factors among patients with SjD. Our results demonstrate a notably high prevalence of frailty (27%) and pre-frailty (49%) within this population. Importantly, elevated CRP levels, higher ESSDAI scores, and increased ESSPRI scores emerged as independent predictors of frailty. Our study cohort consisted of hospitalized SjD patients, who may exhibit higher disease severity and systemic inflammation than community-dwelling patients. Therefore, frailty prevalence and associated risk factors observed here may not fully generalize to all patients with SjD. To our knowledge, this study is the first to systematically characterize the prevalence and determinants of frailty in SjD patients, offering critical insights into the long-term disease burden and potential targets for intervention in this vulnerable cohort.

Our observed frailty prevalence of 27% and pre-frailty prevalence of 49% among patients with SjD substantially exceed those observed in the general community-dwelling elderly population (frailty: 12%, pre-frailty: 46%) (20). When compared to other rheumatic diseases, the prevalence of frailty in SjD patients appears comparably elevated (e.g., 17% in polymyalgia rheumatica [PMR] (21), 35.1% in SSc (22), and 16.6% in rheumatoid arthritis [RA] (22)). Recent evidence from large-scale studies confirms that frailty is more prevalent in patients affected by rheumatic diseases than in healthy controls, regardless of age, and is associated with high disease activity largely due to chronic inflammation (23). These differences likely reflect the combined effects of disease-specific pathophysiological mechanisms, levels of systemic inflammation, treatment strategies, and demographic variations across autoimmune diseases.

Our study revealed that frail SjD patients exhibited significantly higher CRP levels, higher ESSDAI scores, higher ESSPRI scores, and longer disease duration compared to non-frail patients. These findings suggest that chronic inflammation and high disease activity play a crucial role in the development of frailty in the SjD population (7, 24). However, it is important to acknowledge that SjD is inherently a chronic inflammatory autoimmune disorder characterized by persistent immune activation and systemic inflammation (1). This raises the question of whether inflammation serves as a true independent risk factor for frailty or represents an integral component of the disease pathophysiology that inevitably contributes to frailty development. In our multivariable analysis, elevated CRP levels remained an independent risk factor even after adjusting for disease activity measures (ESSDAI and ESSPRI), suggesting that systemic inflammation may have effects on frailty development beyond its contribution to disease activity. This finding aligns with the broader understanding that inflammation accelerates the depletion of physiological reserves by promoting protein breakdown, inducing oxidative stress, affecting muscle metabolism, and disrupting neuroendocrine regulation (23, 24). For SjD patients, controlling chronic inflammation through appropriate immunosuppressive therapy could potentially serve as a dual strategy for managing both disease activity and preventing frailty progression (24). The concept of inflammaging offers a mechanistic explanation. IL-6 and TNF-α are central cytokines in chronic low-grade inflammation and have been linked to functional decline and frailty in rheumatic diseases (24). Previous studies have shown that frail individuals exhibit higher circulating IL-6, TNF-α, and CRP, and IL-6 in particular remains consistently elevated across age-related conditions (25, 26). Additionally, TNF-α promotes muscle proteolysis and mitochondrial dysfunction, contributing to sarcopenia-related frailty (27). In SjD, persistent immune activation may sustain elevated IL-6 and TNF-α, facilitating muscle weakness and reduced physiological reserve. Future studies incorporating cytokine measurements could further clarify their role in the inflammation-frailty pathway.

ESSDAI and ESSPRI scores were identified as independent risk factors for frailty in our SjD cohort. The prevalence of frailty in SjD patients with high disease activity (42.5%) was significantly higher than that in the low activity group (17.1%), demonstrating a clear dose-response relationship. This finding is particularly relevant for SjD, as the disease often involves multiple organ systems with varying degrees of severity (1). The persistent inflammatory responses and immune system overactivation characteristic of active SjD may accelerate the depletion of physiological reserves, thereby increasing frailty risk (28). Similar relationships have been validated in other autoimmune conditions such as RA and SLE, where controlling disease activity significantly reduces frailty risk (29, 30). Given the dynamic nature of frailty and the potential for improvement with targeted interventions, our findings suggest that aggressive management of SjD disease activity may serve as an effective strategy to prevent or potentially reverse frailty progression in this specific patient population (23).

This study also observed a positive correlation between age and frailty. The prevalence of frailty in patients aged 60–86 years was significantly higher compared to those aged 24–44 years, consistent with the consensus that frailty is an age-related syndrome (31). However, in the multivariable analysis, age was not identified as an independent risk factor, suggesting that disease activity and chronic inflammation may have more direct effects on frailty risk than age itself (29). Notably, even among patients younger than 60 years, the prevalence of frailty (18.9%) was higher than that reported for elderly individuals in the general community (11%) (31), indicating that immune dysregulation and chronic inflammation associated with SjD may lead to “premature frailty” in younger patients (29, 32). This phenomenon of accelerated aging in rheumatic diseases has been increasingly recognized, with inflammaging—chronic low-grade inflammation that develops with age—serving as a common driver of age-related frailty (24).

Analysis of individual frailty components revealed that the most common manifestations in our SjD cohort were weakness (56.1%) and exhaustion (54.4%), which differs from the general elderly population where low physical activity (22%) and slowness (20%) are most prevalent (6). This pattern is consistent with frailty characteristics observed in other autoimmune rheumatic diseases such as PMR, RA, and SLE (11, 21, 33), suggesting shared pathophysiological mechanisms. The predominance of weakness and fatigue in SjD patients can be directly attributed to the disease’s pathophysiology, as fatigue and pain are core symptoms affecting approximately 80% of SjD patients (34). Although certain components of the Fried frailty phenotype, such as exhaustion, may appear clinically similar to SjD-related symptoms, the measurement constructs differ. Weakness was assessed using objective grip strength testing rather than subjective perception, and exhaustion was evaluated using standardized CES-D items reflecting psychological fatigue rather than disease-specific symptoms. Additionally, ESSPRI was included in the multivariable model to account for patient-reported fatigue and pain, reducing potential measurement overlap. Importantly, patients with fibromyalgia were excluded based on diagnostic criteria, minimizing confounding from fibromyalgia-related fatigue or pain. The underlying mechanisms in SjD include chronic inflammatory responses, sleep disturbances due to sicca symptoms, autonomic dysfunction, and psychological factors such as depression and anxiety (34). Additionally, the chronic nature of glandular dysfunction leading to persistent dryness may contribute to sleep disruption and subsequent daytime fatigue. Although peripheral arthritis could potentially affect grip strength measurements, only 17.2% of our cohort exhibited peripheral arthritis, minimizing this potential confounding factor. The dominance of weakness and exhaustion in our SjD patients likely reflects the cumulative impact of systemic inflammation, symptom burden, and disease-specific complications rather than the typical age-related decline seen in the general population.

This study found that corticosteroid use (both dosage and exposure duration) was significantly higher in the frail group compared to the non-frail group in univariate analysis. However, these factors did not retain statistical significance in the multivariate analysis, suggesting potential confounding by disease severity and activity. This finding requires careful interpretation in the context of SjD management. Corticosteroids in SjD are typically reserved for managing severe systemic manifestations or organ involvement, indicating that patients receiving higher doses or longer treatment courses likely have more severe disease (1). The relationship between corticosteroids and frailty in SjD patients may thus be bidirectional: while corticosteroids are prescribed for more severe disease (which itself increases frailty risk), long-term corticosteroid use can independently contribute to frailty development through mechanisms including muscle atrophy, bone loss, metabolic disturbances, and increased infection risk (23, 35). The loss of significance in multivariate analysis suggests that disease activity measures (ESSDAI/ESSPRI) may capture the underlying disease severity that necessitates corticosteroid use. Future longitudinal studies in SjD patients are needed to disentangle the complex relationships between disease severity, treatment choices, and frailty development, and to determine optimal corticosteroid management strategies that balance disease control with frailty prevention.

Recognizing frailty risk in patients with SjD may also support more personalized treatment decision-making, as frailty has important implications for medication tolerance, functional outcomes, and long-term disease management.

## Limitations

This single-center study recruited hospitalized SjD patients, which likely led to an overestimation of frailty prevalence due to higher disease severity compared to community or outpatient populations. Therefore, findings may not be generalizable beyond the inpatient setting, and selection bias might have influenced the associations observed. The cross-sectional design limits causal inference; it remains unclear whether high disease activity causes frailty or vice versa, especially regarding patient-reported symptoms like ESSPRI. Additionally, the relatively small sample size resulted in a low events-per-variable ratio in multivariate analyses, potentially affecting model stability. Frailty components relying on self-report, such as unintentional weight loss and exhaustion, are subject to recall bias, possibly leading to misclassification. Finally, the study lacked a healthy control group, as it was designed as a hospital-based cross-sectional investigation. This limits the ability to directly contextualize the prevalence of frailty in SjD relative to the general population. The exclusive inclusion of hospitalized patients may have overestimated the prevalence of frailty compared with outpatient or community SjD populations. Moreover, Although the EPV ratio in our multivariable model is at the lower end of the recommended range, sensitivity analyses confirmed that the identified associations remain robust. Despite these limitations, the study offers valuable insights into frailty in SjD, highlighting the need for larger, longitudinal studies.

## Conclusion

In conclusion, this study clearly identifies the high prevalence of frailty in SjD patients and confirms that CRP, ESSDAI, and ESSPRI are independent risk factors for frailty. The findings suggest that frailty assessment should be emphasized in clinical practice, particularly in patients with high disease activity, high symptom burden, and elevated inflammation levels. Early intervention based on frailty evaluation may help mitigate the impact of these factors on clinical outcomes.

## Data Availability

The original contributions presented in the study are included in the article/**Supplementary Material**. Further inquiries can be directed to the corresponding authors.
