# Supplementary material for: Frailty and associated risk factors in patients with Sjögren’s disease: a cross-sectional study
Source: Front Immunol. 2025 Dec 19;16:1689685. doi: 10.3389/fimmu.2025.1689685 (PMC12757420; doi:10.3389/fimmu.2025.1689685)
Supplement: Supplementary file 1 [file Table1.docx]

**Table S1. Prevalence of Fried Frailty Components and Categorization**

| **Components** | **Participants, n (%)** |
| --- | --- |
| Weight loss | 48 (27) |
| Exhaustion | 98 (54) |
| Inactivity | 57 (32) |
| Slowness | 47 (26) |
| Weakness | 101 (56) |
| **Categorization** |  |
| Robust | 43 (24) |
| Pre-frail | 89 (49) |
| Frail | 48 (27) |

Fried frailty components: The components of frailty as defined by the Fried Frailty Phenotype including weight loss exhaustion inactivity slowness and weakness. Categorization: Based on the number of frailty components present participants are categorized as robust pre-frail or frail.

**Table S2. Distribution of Frailty Phenotype Across Different Age Groups**

| Frailty phenotype | 24–44 years (n = 28) n (%) | 45–59 years (n = 62) n (%) | 60–86 years (n = 90) n (%) | P value |
| --- | --- | --- | --- | --- |
| Robust | 12 (42.9) | 17 (27.4) | 14 (15.6) | <0.001 |
| Pre-frail | 13 (46.4) | 31 (50.0) | 45 (50.0) |  |
| Frail | 3 (10.7) | 14 (22.6) | 31 (34.4) |  |

Pairwise comparisons: 24–44 years vs 45–59 years Padj = 0.135; 24–44 years vs 60–86 years Padj = 0.002; 45–59 years vs 60–86 years Padj = 0.063. P value < 0.05 indicates statistical significance for frailty distribution across age groups. Padj adjusted P value using the Bonferroni correction for multiple comparisons. Jonckheere–Terpstra test was used to analyze the trend in frailty phenotype distribution across age groups.

**Table S3. Distribution of Frailty Phenotype Across Different ESSDAI Activity Groups**

| Frailty phenotype | ≤4 (n = 76) n (%) | 5–13 (n = 64) n (%) | ≥14 (n = 40) n (%) | P value |
| --- | --- | --- | --- | --- |
| Robust | 26 (34.2) | 12 (18.8) | 5 (12.5) | <0.001 |
| Pre-frail | 37 (48.7) | 34 (53.1) | 18 (45.0) |  |
| Frail | 13 (17.1) | 18 (28.1) | 17 (42.5) |  |

Pairwise comparisons: ≤4 vs 5–13 Padj = 0.039; 5–13 vs ≥14 Padj = 0.194; ≤4 vs ≥14 Padj = 0.001. P value < 0.05 indicates statistical significance for frailty phenotype distribution across ESSDAI groups. Padj adjusted P value using the Bonferroni correction to control for multiple comparisons. Jonckheere–Terpstra test was used to analyze the trend in frailty phenotype distribution across ESSDAI activity groups. ESSDAI categories: ≤4 (low activity) 5–13 (moderate activity) ≥14 (high activity).

**Table S4. Demographic and Clinical Characteristics of SjD Patients by Fried Frailty Classification**

| Characteristics | Robust (n=43) | Pre-frail (n=89) | Frail (n=48) | P value |
| --- | --- | --- | --- | --- |
| Age, years median (IQR) | 55.0 (41.0 63.0) | 60.0 (50.0 68.0) | 64.0 (54.0 70.0) | 0.002 |
| Sex, female n (%) | 41 (95.3) | 84 (94.4) | 42 (87.5) | 0.292 |
| BMI, (kg/m²) mean (SD) | 23.7 (2.7) | 22.3 (3.5) | 22.6 (4.1) | 0.063 |
| Ever smoked, n (%) | 3 (7.0) | 6 (6.7) | 4 (8.3) | 0.931 |
| Ever used alcohol, n (%) | 3 (7.0) | 6 (6.7) | 5 (10.4) | 0.716 |
| Living alone, n (%) | 5 (11.6) | 8 (9.0) | 3 (6.3) | 0.698 |
| Low education, n (%) | 23 (53.5) | 60 (67.4) | 38 (79.2) | 0.034 |
| Disease duration months, median (IQR) | 36.0 (12.0 60.0) | 60.0 (18.0 138.0) | 84.0 (39.0 171.0) | <0.001 |
| ESR, (mm/h) median (IQR) | 14.0 (7.0 25.0) | 19.0 (10.0 37.5) | 21.5 (10.3 34.8) | 0.045 |
| CRP, (mg/L) median (IQR) | 2.0 (1.0 3.0) | 2.0 (1.6 4.0) | 3.0 (2.0 7.0) | 0.009 |
| RF+, n (%) | 22 (51.2) | 42 (47.2) | 20 (41.7) | 0.657 |
| ESSDAI, median (IQR) | 3.0 (0 6.0) | 5.0 (2.0 11.0) | 9.5 (4.0 16.5) | <0.001 |
| ESSPRI, median (IQR) | 3.0 (1.0 4.0) | 5.0 (3.0 7.0) | 5.0 (4.0 7.0) | <0.001 |
| Prednisolone-equivalent dose, mg/day median (IQR) | 0 (0 15.0) | 0 (0 15.0) | 8.8 (0 30.0) | 0.037 |
| Glucocorticoid exposure duration, days median (IQR) | 0 (0 1) | 0 (0 1) | 1.0 (0 204.3) | 0.040 |
| cDMARDs use, n (%) | 40 (93.0) | 80 (89.9) | 44 (91.7) | 0.942 |
| Charlson Comorbidity Index, median (IQR) | 1 (0 1) | 1 (0 1) | 1 (0 2) | 0.137 |

**Table S5. Pairwise Comparisons of Frail Phenotype (Adjusted P Values with Bonferroni Correction)**

| Variables | Robust vs Pre-frail | Robust vs Frail | Pre-frail vs Frail |
| --- | --- | --- | --- |
| Age | 0.036 | 0.002 | 0.436 |
| Low education | 0.363 | 0.027 | 0.438 |
| Disease duration | 0.029 | <0.001 | 0.168 |
| ESR | 0.115 | 0.056 | 1.000 |
| CRP | 1.000 | 0.014 | 0.029 |
| Glucocorticoid exposure duration | 1.000 | 0.049 | 0.133 |
| Prednisolone-equivalent dose | 1.000 | 0.035 | 0.237 |
| ESSDAI | 0.049 | <0.001 | 0.103 |
| ESSPRI | <0.001 | <0.001 | 0.424 |

Data were compared using the χ² or Fisher's exact test for categorical variables and the Kruskal-Wallis test for continuous variables. Values in bold indicate statistical significance (P < 0.05). For variables with significant results pairwise comparisons were performed with Bonferroni correction applied to adjust for multiple comparisons. Adjusted P values (Padj) are reported.

**Table S6. Collinearity Diagnostics for Variables in the Logistic Regression Model**

| Variable | VIF | Tolerance |
| --- | --- | --- |
| age | 1.109 | 0.902 |
| CRP | 1.041 | 0.961 |
| ESSDAI | 1.020 | 0.980 |
| ESSPRI | 1.092 | 0.916 |

VIF variance inflation factor. VIF values and tolerance values are used to assess potential multicollinearity issues. A VIF greater than 10 or a tolerance value below 0.1 would indicate problematic multicollinearity. All variables show VIF values well below 10 and tolerance values above 0.1 indicating no significant multicollinear

**Table S7. Multivariable Analysis of Risk Factors Associated With the Frailty Phenotype in Patients With SjD.**

| Variables | _a2_OR (95% CI) | P value | _a1_OR (95% CI) | P value |
| --- | --- | --- | --- | --- |
| Age, years | 1.016 (0.982-1.051) | 0.365 | 1.012 (.977-1.048) | 0.521 |
| Low education |  |  | 1.605 (0.652-3.950) | 0.303 |
| Disease duration | 1.001 (0.997-1.005) | 0.616 | 1.001 (0.997-1.005) | 0.655 |
| CRP, mg/L | 1.077 (1.016-1.142) | 0.012 | 1.079 (1.018-1.144) | 0.010 |
| ESSDAI | 1.081 (1.026-1.139) | 0.003 | 1.078 (1.023-1.136) | 0.005 |
| ESSPRI | 1.263 (1.055-1.511) | 0.011 | 1.249 (1.044-1.495) | 0.015 |

_a1_OR: Model adjusted for age, disease duration, C-reactive protein (CRP), ESSDAI, and ESSPRI.

_a2_OR: Model adjusted for age, low education, disease duration, C-reactive protein (CRP), ESSDAI, and ESSPRI.
